# A novel arginine methylation-associated lncRNA signature effectively predicts prognosis in breast cancer patients

**DOI:** 10.3389/fonc.2024.1472434

**Published:** 2024-10-01

**Authors:** Changli Wang, Shuaishuai Wu, Yanran Hu, Jingjing Wang, Kun Ru, Miaoqing Zhao

**Affiliations:** ^1^ Department of Pathology, Shandong Cancer Hospital and Institute, Shandong First Medical University and Shandong Academy of Medical Sciences, Jinan, China; ^2^ Department of Neurosurgery, Shandong Cancer Hospital and Institute, Shandong First Medical University and Shandong Academy of Medical Sciences, Jinan, China

**Keywords:** breast cancer, arginine methylation, lncRNA, prognosis, cancer biomarkers

## Abstract

Breast cancer (BC) is a disease highly associated with epigenetic modification, and arginine methylation is particularly important in its genetic regulation. However, the role of arginine methylation related lncRNAs in breast cancer has not been studied. First, we identified the related lncRNAs (from TCGA database) according to the differentially expressed genes related to arginine methylation in breast cancer. Then the lncRNAs related to protein arginine methylation were obtained by regression analysis, and the risk score model was constructed. Finally, the cell experiment and subcutaneous tumor model verified that the arginine methylation related lncRNA z68871.1 in the model had a significant effect on the proliferation and invasion of breast cancer cells. In conclusion, we successfully constructed an arginine methylation related lncRNA model, which has strong predictive ability. At the same time, this study provides an experimental basis for exploring the mechanism of arginine methylation in BC and helps to find new biomarkers of BC.

## Introduction

Breast cancer (BC) is an incurable disease that causes great harm to women (1). Despite considerable progress in early diagnosis and individual therapy over the past decades, drug resistance, recurrence, metastasis, and cancer heterogeneity remain important causes of cancer-related deaths and are significant issues hindering the successful management of BC (2). Individualized specific targeted therapies are receiving increasing attention in clinical work. Therefore, it is necessary to explore new biomarkers with diagnostic and therapeutic significance.

Arginine methylation is a common post-translational modification of proteins (3), similar to phosphorylation and ubiquitination (4), it plays a vital role in cell biology, where it can affect protein function, stability, and localization, as well as participate in cancer-related epigenetics and signaling, RNA metabolism, and DNA repair (5–7). Recent research has demonstrated that arginine methylation plays a role in the progression of breast cancer (8, 9). Therefore, identifying key regulatory factors of arginine methylation is crucial for basic research and diagnosis and treatment of tumors.

Currently, long non-coding RNA (lncRNA) plays an important role in a variety of genetic material regulation processes (10), including proliferation, apoptosis, metastasis, metabolism and drug resistance (11, 12). In addition, the emergence of genomics and next-generation sequencing technologies has provided new evidence for the impact of lncRNAs on gene regulation (13). lncRNAs, as one of the critical regulators of genetic material, affect the stability and various modifications of genetic material by regulating nuclear structure and transcription (14). With further research, it was discovered that lncRNA boosts tumor progression by regulating arginine methylation and have the potential to predict the deterioration of various cancer prognoses (15–17). Therefore, in cancer research, it is important to identify key lncRNAs closely related to arginine methylation. It is important in the treatment and prognosis evaluation of cancer.

Our study conducted an in-depth survey of the dataset containing lncRNA expression profiles in BC from TCGA database. Our screening process focuses on identifying lncRNAs with prognostic significance associated with arginine methylation. Therefore, we identified 8 different lncRNA signals associated with arginine methylation prognosis, which may accurately predict the survival outcomes of cancer patients.

## Materials and methods

### Data source

All data (including age, gender, histologic grade, OS time, survival status and gene expression) were from the TCGA database. After data collation, 1096 breast cancer and 112 standard tissue samples were finally obtained (excluding data with survival time less than 30 days and unrecorded data collection).

### Identification of arginine methylation-related lncRNAs

The arginine methylation-related genes included in the study were obtained from the GeneCards database. First, we investigated the differentially expressed genes (DEGs, (|log2 FC|> 1). Next, Cox regression analysis was performed to identify key LncRNAs as prognostic features of BC.

### Predictive risk mode

We constructed a risk score (RS) based on 8 predictive features. $risk\;score=\sum_{i=1}^{n}coef\;AMT\;LncSigi\;X\;EXP\;AMT\;LncSigi$ .

The “Coef AMT LncSigi” in the formula represents the value of the multifactorial regression coefficient, and the “EXP AMT LncSigi” indicates the expression of lncRNA.

### Immune cell function and drug analysis

Immune cell activity and pathways were calculated for each sample using single-sample GSEA (ssGSEA), and then the proportion of infiltrating immune cells in the tumor samples was assessed and immune-related drugs were evaluated and predicted using the “pRRophetic” R package.

### Cell transfection

We purchased two cell lines (MCF-7 and MDA-MB-231) from the Cell Bank of the Chinese Academy of Sciences. Cells with good growth conditions were taken and digested with trypsin. Then, the cells were inoculated in 6-well plates(1×10^5^) and placed in the 5% CO2 cell culture incubator for lentiviral transfection experiments until the cell confluence was 20~30%. Lentiviral expression vectors with Z68871.1 sequence were purchased from Shanghai Jikai Gene Medical Technology Co. According to the instructions of the reagent supplier, cells were infected with packaged lentivirus.

### Clone formation experiment and radiosensitivity analysis

MCF-7, MDA-MB-231 experimental group and control group were digested with trypsin and resuspended as a single cell suspension, respectively; 1000 cells/well were put into the corresponding cells in 6-well Petri dishes and placed in the cell incubator for two weeks. The number of colonies was counted for analysis. Colony formation was used to assess the proliferative capacity of the cells. At the same time, in order to further understand the effect of z68871.1 on the radiosensitivity of breast cancer cells, MCF-7 cells were irradiated with doses of 0Gy, 3Gy and 6Gy, and the cell survival fraction was observed by colony formation assay.

### Transwell invasion experiment

Transwell chambers (Corning Costar, 8 μm) were primed with Matrigel matrix gel. The transfected control and experimental cells were resuspended with an FBS-free DMEM medium. After 24 hours of cultivation, we fixed the cells with 4% PFA, stained and counted them.

### CCK-8 cell proliferation experiment

Cells in the logarithmic growth phase were taken to prepare a single-cell suspension. The cells were counted with a glass plate, and the cell concentration was 2 × 104/ml. Take the cell culture plate, and each group of cells is set at 2000/100 µ l/well, with 3 parallel samples in each group, and add the whole culture medium. Continue to cultivate in the incubator. CCK8 reagent (10 µL/well) was added to 96 well plates after 0, 24, 48, 72 and 96 hours, respectively. Cell-free medium wells can be used as blank control. Continue to incubate in the cell incubator for 1-4 hours. The microplate reader measured the absorbance value of 450nm. Draw the growth curve of each group of cells.

### Subcutaneous tumor formation experiments in mice

Five-week-old female nude mice (Guangdong Pharmachem Biotechnology Co., Ltd., Guangzhou, China) were divided into two groups. MCF-7 cells were divided into the NC and Z68871.1-ox groups, respectively. The number of cells injected subcutaneously into each mouse was 1×106, and weekly live imaging was performed to record the growth of tumors. This study was approved by the ethics committee of the Cancer Hospital Affiliated with Shandong First Medical University.

### Statistical analysis

R software (https://www.r-project.org/) was used for all analyses. The results of clone formation and transwell invasion experiments were analyzed using ImageJ software, and GraphPad Prism 8.0 was used to analyze the statistical significance of the difference between the two groups. Correlation coefficients were examined using the Pearson correlation test. P < 0.05 was considered significant.

## Results

### Screening of arginine methylation-associated lncRNAs

**Figure 1:**
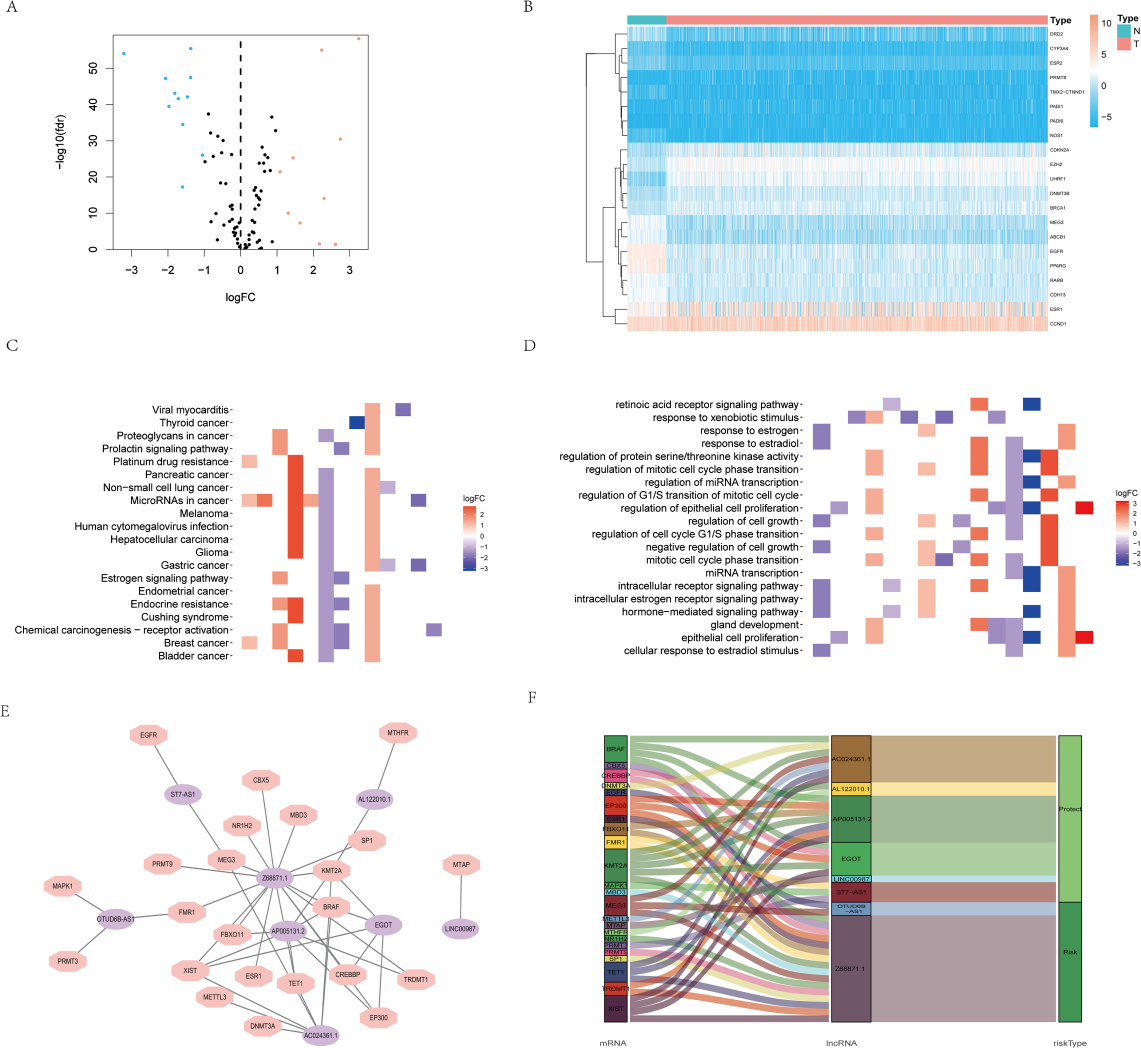
Screening of arginine methylation-related lncRNAs. **(A)** Volcano plot of 10 up-regulated and 11 down-regulated DEGs in BC. **(B)** Heatmap of 21 protein arginine methylation-associated DEGs. **(C)** KEGG analysis of protein arginine methylation-associated DEGs. **(D)** GO analysis of protein arginine methylation-associated DEGs. **(E)** The co-expression network of prognostic protein arginine methylation-associated lncRNAs. **(F)** Sankey diagram of prognostic protein arginine methylation-associated lncRNAs. N, normal; T, tumor.

Based on the GeneCards database, 111 arginine methylation-associated genes were obtained, and the screening criterion was a correlation score of >10 for protein-coding genes (**Supplementary Data Sheet 1**). Subsequently, 21 arginine methylation-associated DEGs were obtained by differential analysis of BC and normal breast tissues (**Figures 1A, B**). Enrichment analysis demonstrated that the above genes were enriched in cell growth and division, hormone and immune, cytokine receptor and tumor-related biological function signaling pathways (**Figures 1C, D**). Then, 1199 arginine methylation-associated lncRNAs (**Supplementary Data Sheet 2**) were screened by arginine methylation-associated DEGs. Univariate Cox regression screened 48 prognostic-related lncRNAs (**Supplementary Material 3**). Multivariate Cox regression identified 8 lncRNAs with the best predictive correlation: AL122010.1, OTUD6B-AS1, EGOT, AP005131.2, Z68871.1, AC024361.1, LINC00987, ST7-AS1 (**Table 1**), and we further visualized the correlation between lncRNAs and mRNAs using Cytoscape (**Figures 1E, F**), showing that Z68871.1, AP005131.2 and AC024361.1 were most closely associated with mRNAs.

**Table 1:** Prognostic arginine methylation-associated lncRNAs.

id	coef	HR	HR.95L	HR.95H	pvalue
AC024361.1	-0.896229	0.4081056	0.1720781	0.9678756	0.0419446
LINC00987	-0.576666	0.5617683	0.3409932	0.9254839	0.0235749
Z68871.1	0.5422145	1.7198112	0.8856762	3.3395393	0.1092875
AL122010.1	-0.763817	0.4658849	0.2552217	0.8504324	0.0128608
OTUD6B-AS1	0.5714034	1.7707504	1.0852829	2.8891607	0.02216
ST7-AS1	-0.717528	0.4879571	0.2257422	1.0547524	0.0680878
AP005131.2	-0.497396	0.6081124	0.3017806	1.2253959	0.1641129
EGOT	-0.411083	0.662932	0.4442858	0.9891805	0.0440894

### Construction of predictive risk model and internal validation

**Figure 2:**
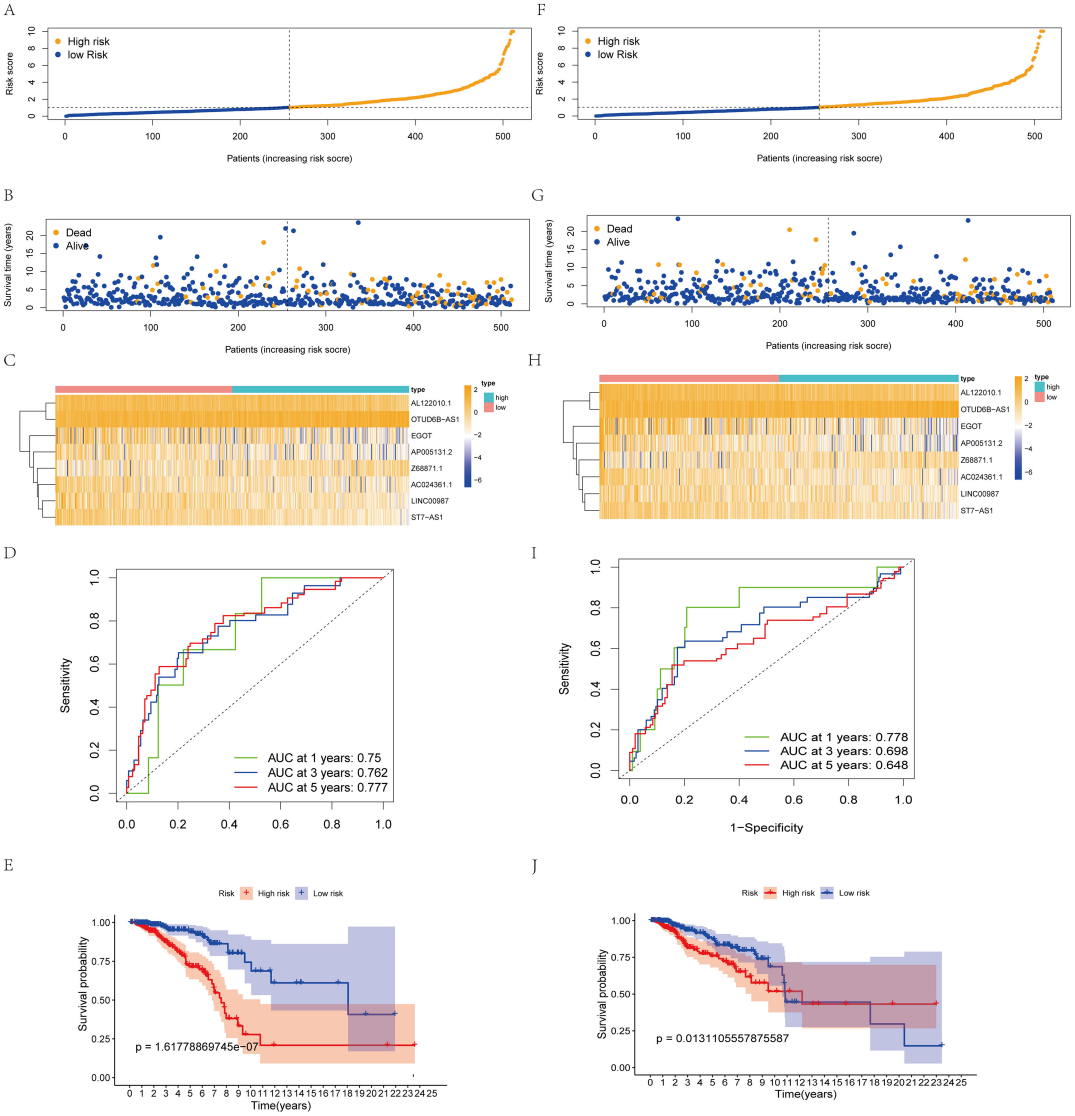
Construction and validation of the prognostic protein arginine methylation-associated lncRNAs signature for survival prediction. **(A-E)** Distribution of RS; survival time and status of patients; heatmap of protein arginine methylation-associated lncRNAs of RS; ROC curve; K-M curve for training Data. **(F-J)** Distribution of RS; survival time and status of patients; heatmap of protein arginine methylation-associated lncRNAs of RS; ROC curve; KM curve for validation Data.

**Figure 3:**
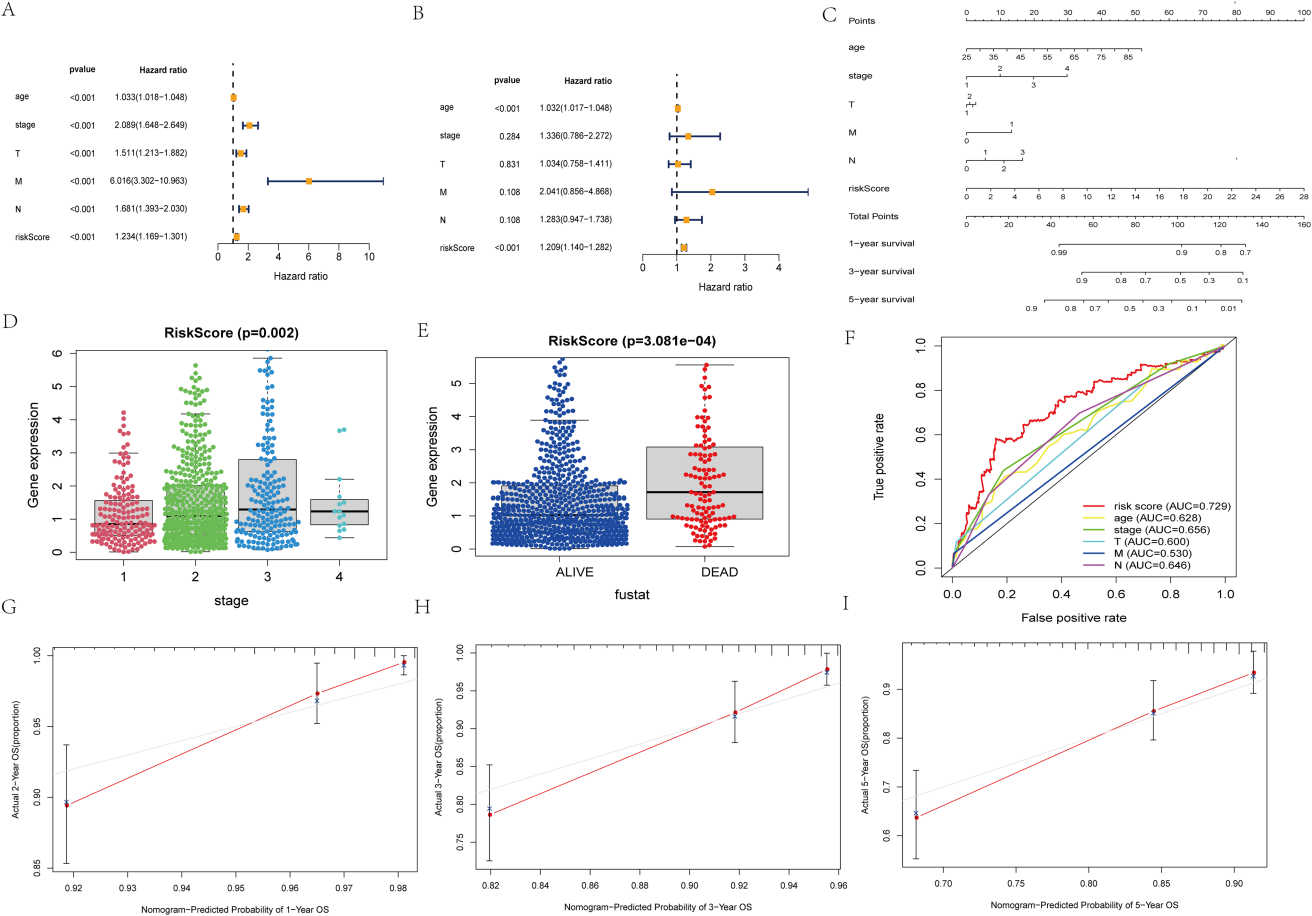
Independent prognosis analysis of risk score. **(A)** Univariate COX Forest plot of risk score. **(B)** Multivariate COX Forest plot of risk score. **(C)** A nomogram based on prognostic features. **(D, E)** Relationship between stage staging, survival status and risk score of patients. **(F)** ROC curve of risk score and clinical characteristics. **(G–I)** Calibration plots of the nomogram for predicting the probability of OS at 1, 3, and 5 years.

We used the RS = (0.542214503 × Z68871.1 expression) + (0.571403384 × OTUD6B-AS1 expression) - (0.8962291962× AC024361.1 expression) - (0.576665716 × LINC0098 expression) - (0.76381661 × AL122010.1 expression) - (0.717527719 × ST7-AS1 expression) - (0.497395506 × AP005131.2 expression) - (0.411082836 × EGO expression) to calculate the score of each patient. Patients in the model were categorized into two groups (high- and low-risk groups), and patient scores were positively related to prognosis (**Figures 2A, B**). lncRNA expression levels also showed significant differences (**Figure 3C**). The ROC curves for the model training set data were 0.75 (1 year), 0.762 (3 years), and 0.777 (5 years), and the K-M curves also demonstrated a poor prognosis for patients with high RS (**Figures 2D, E**).

Using the same threshold, the validation set was divided into two groups. Patient distribution was similar to the training set (**Figure 2F**), and high scores were positively associated with worse prognosis (**Figure 2G**). The validation set AUC value and lncRNA expression levels were similar to the training set (**Figures 2H, I**). K-M analysis proved that the model predicted well (p<0.05, **Figure 2, J**).

### Construction of nomogram diagrams and validation of accuracy

The results of unifactorial and multifactorial analyses of clinical characteristics demonstrated that the model could predict patient prognosis independently of other clinicopathologic data (**Figures 3A, B**). Next, we constructed a nomogram diagram based on clinicopathologic data T, N, M grading, and risk score, which was used to calculate survival probabilities for clinical workup (**Figure 3C**). Upon further analysis, the risk model was significantly correlated with patient staging and prognosis (**Figures 3D, E**). The ROC curves suggest that predictive risk models perform best when compared to other clinical data (**Figure 3F**). Calibration curves show good agreement between model predictions and actual conditions (**Figures 3G–I**).

**Figure 4:**
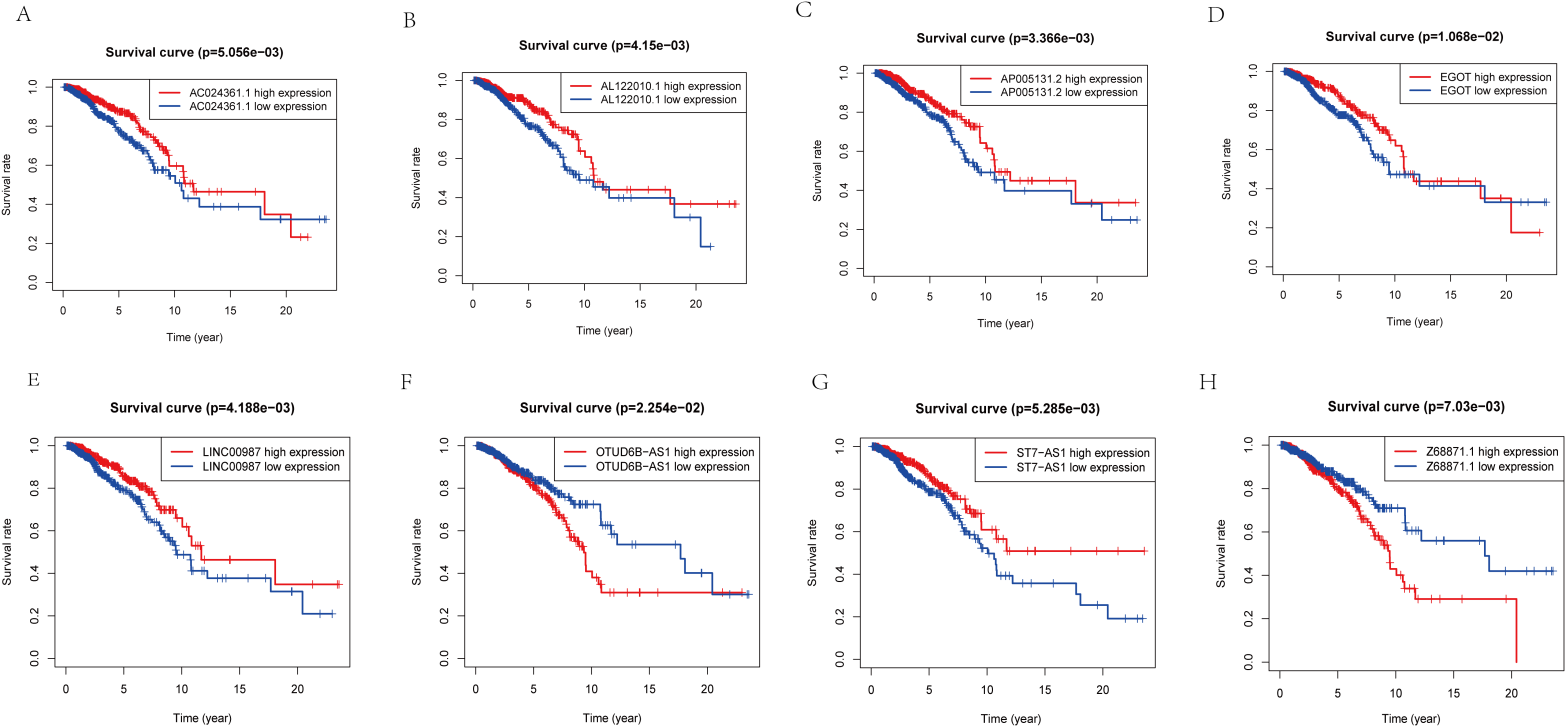
K-M analysis of lncRNA expression level in predictive model and prognosis of patients. **(A)** The relationship between the expression level of AC024361.1 and prognosis. **(B)** The relationship between the expression level ofAL122010.1 and prognosis. **(C)** The relationship between the expression level of AP005131.2 and prognosis. **(D)** The relationship between the expression level of EGOT and prognosis. **(E)** The relationship between the expression level of LINC00987 and prognosis. **(F)** The relationship between the expression level of OTUD6B-AS1 and prognosis. **(G)** The relationship between the expression level of ST7-AS1 and prognosis. **(H)** The relationship between the expression level of Z68871.1 and prognosis.

To further validate the accuracy of the risk model, we analyzed the survival curves of individual genes in the model (**Figures 4A–H**). We found that similar to the multifactorial results, high expression levels of AC024361.1, LINC00987, AL122010.1, EGOT, ST7-AS1, and AP005131.2 were correlated with a better prognosis; on the contrary, high expression levels of OTUD6B-AS1 and Z68871.1 were associated with a poor prognosis of patients.

### Immune cell infiltration and functional analysis

**Figure 5:**
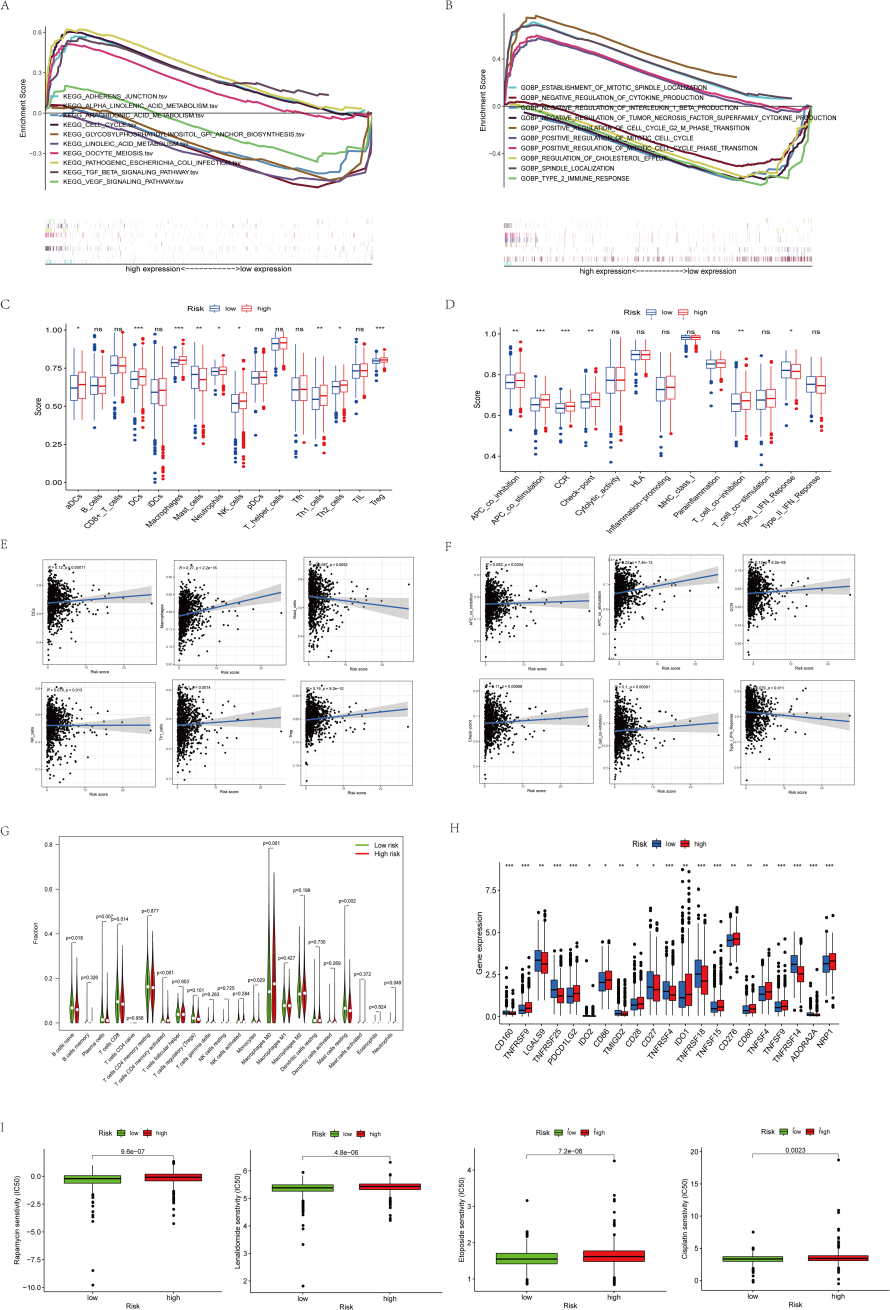
Functional enrichment analysis of 8 prognostic arginine methylation-associated lncRNAs. **(A)** KEGG analysis of 8 prognostic arginine methylation-associated lncRNAs. **(B)** GO analysis of 8 prognostic arginine methylation-associated lncRNAs. **(C, E)** Infiltration level and correlation of 16 kinds of immune cells. **(D, F)** The correlation between the predictive signature and 13 immune-related functions. **(G)** Analysis of immune cell infiltration between high-risk group and low-risk group. **(H)** Analysis of immune checkpoints between high-risk group and low-risk group. **(I)** IC50 of Rapamycin, Lenalidomide, Etoposide, and Cisplatin in high and low-risk groups. (ns *P* > 0.05, *P < 0.05; **P < 0.01; ***P < 0.001.).

We further investigated the biological functions of prognostic factors using GSEA and found that many immune-related pathways and cell metabolism-related pathways were associated with high-risk populations (**Figures 5A, B**). we further performed ssGSEA enrichment analysis and found that in the high-risk populations, most cells (DC cells, Th1 cells, NK cells, Tregs, etc.) were significantly elevated (**Figure 5C**). APC co-stimulation, CCR, T cell co-inhibition, and checkpoint were significantly observed in the high-risk populations (**Figure 5D**). Meanwhile, in order to better understand their correlation, we specifically quantified the relationship coefficients between immune-related pathways and the proportion of immune cells (**Figures 5E–G**). Given the importance of immune checkpoints for BC, we also analyzed 21 common immune checkpoints and found that PDCD1LG2, CD86, IDO1, CD276, TNFSF9, and NRP1 were higher in the high-risk populations (**Figure 5H**). Finally, relevant drug prediction analysis showed that the IC50 values of Etoposide, Rapamycin, Lenalidomide, and Cisplatin were higher in the high-risk populations (**Figure 5I**).

### Overexpression of Z68871.1 enhances proliferation and invasion of BC

**Figure 6:**
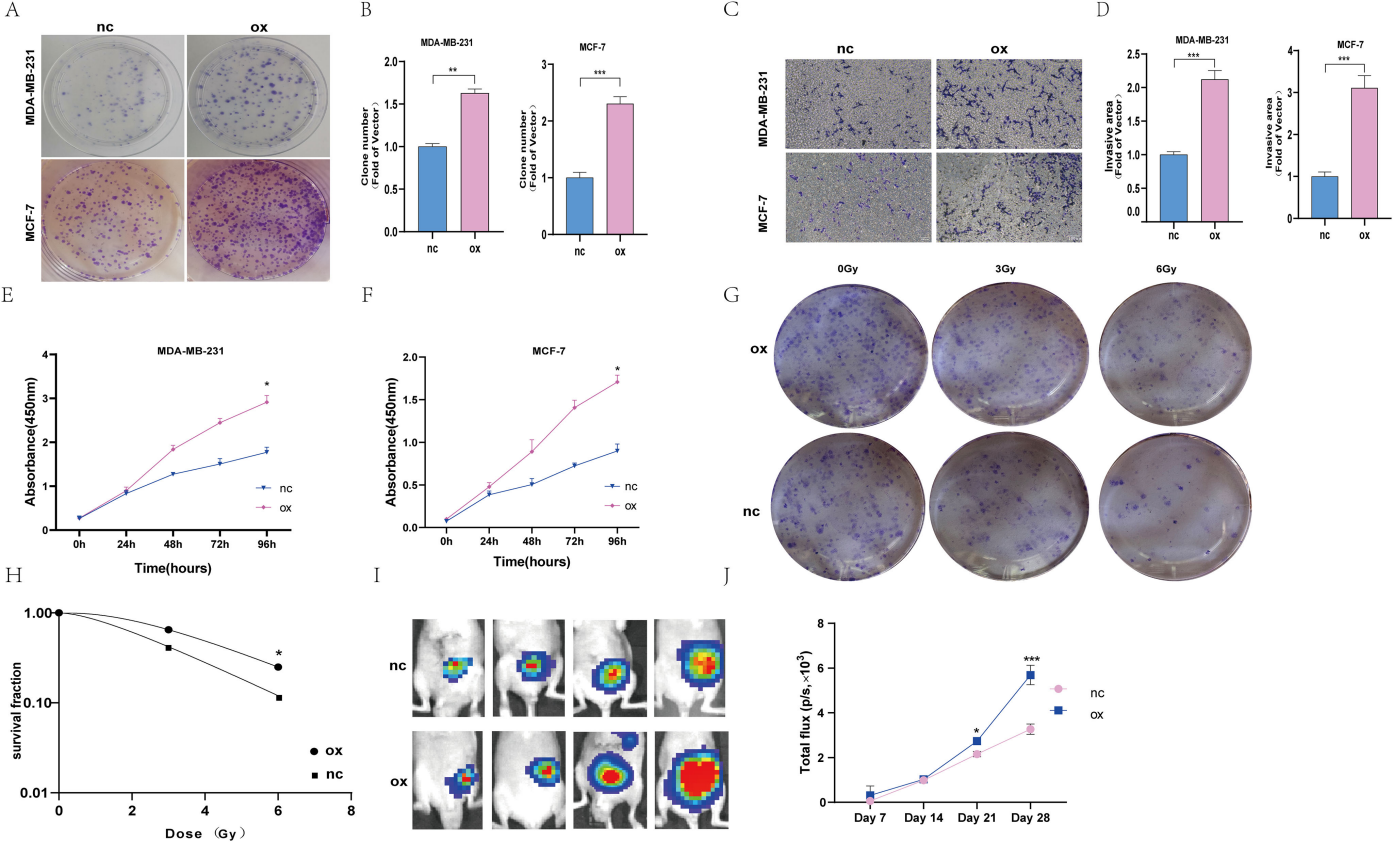
Overexpression of the z68871.1 gene can enhance the proliferation and invasion of MCF-7 and MDA-MB-231 cells. **(A, B)** Cell clones were used to detect the proliferation of MCF-7 and MDA-MB-231 cells. **(C, D)** The transwell method was used to detect the migration and invasion of MCF-7 and MDA-MB-231 cells. **(E, F)** Use CCK-8 method to detect the proliferation ability of MCF-7 and MDA-MB-231 cells. **(G, H)** Cell cloning experiment to detect the effect of Z68871.1 on the radiation sensitivity of MCF-7 cells. **(I, J)** *In vivo* imaging detection of subcutaneous tumour growth in mice. (*P < 0.05; **P < 0.01; ***P < 0.001.).

Among these eight screened genes, most of them, except Z68871.1 and AC024361.1, have sufficient basic experimental studies in cancer. According to our preliminary analysis, the closest association between Z68871.1 and mRNA was found, so it is reasonable to suspect that it plays a crucial role in BC. Therefore, in the following experiments, we investigated whether Z68871.1 plays a role in BC development. First, we overexpressed Z68871.1 in two BC cell lines, and in a cellular functionalization assay, a cell cloning assay showed that overexpression of Z68871.1 enhanced cell viability in BC cells (**Figures 6A, B**). Next, we also examined the invasive ability of cells and found that overexpression of Z68871.1 significantly enhanced their invasive ability (**Figures 6C, D**). Next, we used the CCK8 assay to detect the effect of z68871.1 on cell proliferation. The results showed that in the cell line, there was no significant change in each group at 24 hours. After 72 hours, the proliferation ability of overexpressing z68871.1 cells was stronger (**Figures 6E, F**). In order to further understand the effect of z68871.1 on the radiosensitivity of breast cancer cells and provide useful information for clinical treatment and prognosis, MCF-7 cells were irradiated with doses of 0Gy, 3Gy and 6Gy, respectively. The radiosensitivity of MCF-7 cells was evaluated by plate clone formation assay (**Figures 6G, H**). The survival rate of the nc group at 3Gy and 6Gy doses was lower than that of the ox group (*P <*0.05). The overexpression of z68871.1 attenuated the sensitivity of MCF-7 cells to radiation. Finally, to further test the functional effects of Z68871.1 on BC, we established an animal tumor model to explore the biological effects of Z68871.1. After the mouse tumor model was established, each mouse was subjected to *in vivo* imaging at intervals of 1 weekday to monitor the tumor growth process and plot the growth curve of intracranial meningiomas. The fluorescence values of the intracranial tumors in the two groups of nude mice did not show any significant difference in the first 2 weeks after cell injection, and the fluorescence values of the tumors in the two groups began to change on day 21 (*P <*0.05). Twenty-eight days later, the fluorescence values of tumors overexpressing Z68871.1 were remarkably higher than those of the control group (**Figures 6I, J** *P* < 0.001). These suggest that overexpression of Z68871.1 enhances the proliferation and invasion of BC in a nude mouse tumor model.

## Discussion

Currently, despite the significant advances in the treatment of BC patients, BC accounts for 23% of all cancer-related deaths and poses a major threat to women’s survival (18–20). Therefore, the need to explore new molecular targets and individualized treatments for BC is urgent (21).

Numerous studies have fully demonstrated that arginine methylation is crucial in transcriptional regulation, cell division, cell cycle regulation, DNA repair and substance metabolism in multiple genetic and developmental aspects and critical processes (22). Not only that, arginine methylation has been well studied in cancers (breast, lung, and colon cancers as well as leukemia), and most of the protein arginine methyltransferases have been associated with cancer-related epigenetic and chromatin regulation, transcription, signaling, and metabolic regulation (23). In recent years, research has proved that lncRNAs carry out different functions in the physiological processes of tumors (24–26). Nevertheless, the functions of arginine methylation-associated lncRNAs in BC patients have never been investigated. Therefore, the present study aimed to identify arginine methylation-related lncRNAs, construct a risk model with predictive properties, and characterize the genetic and immune functions of lncRNAs.

Firstly, we screened 21 genes relevant to arginine methylation and then used reliable biological analysis to determine the predictive characteristics of 8 prognostic lncRNAs related to arginine methylation. After analysis, AC024361.1, LINC00987, AL122010.1, EGOT, ST7-AS1, and AP005131.2 have been identified as protective factors; OTUD6B-AS1 and Z68871.1 have been identified as risk factors. Then, GSEA analysis revealed that cell cycle and TGF-β signaling pathways were more marked in the high-risk population. Afterwards, the results regarding TIICs demonstrated that the infiltration abundance of DCs, Th1, NK and Tregs cells was higher in the high-risk population. Finally, basic experiments on lncRNA in the model were conducted again through BC cells and mice experiments, and the results showed that Z68871.1 affected the viability, proliferation and invasion ability of BC cells, and had an impact on the sensitivity of cells to radiation. Therefore, the arginine methylation-associated lncRNAs successfully screened in our work could predict the outcome of BC patients. More importantly, it provides ideas for further exploring the personalized therapeutic value of arginine methylation-related lncRNAs.

In previous studies, lncRNAs were studied, and their biological functions were confirmed. Wang et al. discovered that lncRNA ST7-AS1 was critical in promoting LUAD cell viability, affecting invasive ability and leading to EMT. The mechanism of its carcinogenic activity is mainly achieved by sponge-like miR-181b-5p, thereby relieving the inhibition of KPNA4 (27). In addition, other studies have found that the low expression of ST7-AS1 may change the regulation of the cell cycle, affect the repair ability after DNA damage, and affect the distribution and activity of immune cells in the microenvironment of breast cancer, thus leading to the development of tumors and therapeutic effects (28). Li et al.’s experimentation has shown that overexpression of OTUD6B-AS1 can inhibit the malignant biological characteristics of thyroid cancer cells and is achieved by targeting miR-183-5p (29). However, there are also studies that overexpression of OTUD6B-AS1 can promote tumor progression, such as lncRNA OTUD6B-AS1 promoting malignant transformation of liver cancer cells through Wnt/β-catenin signaling (30). The biological function of LINC00987 has also been studied in lung cancer. Specifically, the progression of lung adenocarcinoma is induced by the downregulation of low methylation related LINC00987, which inhibits phosphorylation-mediated SND1 degradation (31). AL122010.1 and AP005131.2 have previously been reported as immune and metabolic prognostic features of breast cancer (32, 33). AC024361.1 can serves as a risk model of autophagy-associated lncRNA composition (34), but this study did not demonstrate its function experimentally. Studies on EGOT have found that the expression of EGOT is negatively correlated with the survival of breast cancer patients, which is mainly caused by the inactivation of lncRNA EGOT through the Hedgehog pathway (35). Similarly, EGOT research in gastric cancer also found that the loss of EGOT led to the down-regulation of Hedgehog signaling pathway, and could inhibit the proliferation function by preventing the G1 phase cycle process of GC cells (36). Although Z68871.1 has recently been reported as a methyladenosine-modified lncRNA that can predict outcomes for BC patients (37), Zhao et al. did not conduct basic experiments to verify this finding in their study. Therefore, our functional research on Z68871.1 in cells and animals provides relevant evidence for their conclusion to some extent.

Next, we conducted GSEA analysis further to explore other functions of lncRNA models in BC. KEGG analysis shows that high-risk populations are mainly enriched in pathways closely concerned with cell cycle. Functional analysis showed that DEGs were mainly enriched in immune pathways such as anti-tumor factor action, and negative interleukin blockade. Based on the above analysis, we found a correlation between the predictive factors in the model and tumor immunity. Therefore, further study of immune cells and pathways showed that DCs, Th1 cells, NK cells, Tregs, etc. have a higher proportion in high-risk populations, and CCR, T cell co inhibition, and checkpoints are more significant. Previous experimentations have shown that the proportion of different immune cells in BC can affect the prognosis of patients (38). Moreover, it was found that M2 macrophages release cytokines that promote tumor growth. These factors not only stimulate tumor cell proliferation and angiogenesis but also promote tumor metastasis and regulate the extracellular matrix, leading to poor prognosis in BC patients (39). Our research findings are consistent with the appeal’s conclusions, further confirming and strengthening the reliability of our risk prediction model in terms of predictive ability. Finally, our research on immune control points and immune-related drugs aims to explore new directions for immunotherapy of BC. Fortunately, many of the latest experimental results have fully confirmed that the medicines we predicted have achieved remarkable results in improving the poor outcomes of BC patients and animal models of breast cancer (40, 41). This not only validates our research but also brings new hope and strategies for breast cancer patients to provide more accurate and personalized treatment.

This study successfully constructed a prediction model based on high-throughput sequencing data and verified the effectiveness of the model through basic experiments. However, there are still some limitations, such as the need for more sequencing data and prospective studies to verify the accuracy of the model. In addition, the link between z68871.1 and arginine methylation has not been established in this study, and the specific mechanism and function of lncRNA and arginine methylation still need to be further explored, and more basic experiments are needed to help study how z68871.1 promotes the proliferation and invasion of breast cancer cells through arginine methylation. In the future, we will conduct in-depth studies on arginine methylation-related lncRNAs and verify the prediction accuracy of this model through more samples and experiments so as to promote its application in clinical work.

In summary, we have successfully identified lncRNAs with significant predictive value in the process of arginine methylation and established a highly accurate prediction model based on them. And this model not only provides a new perspective for the development of cancer treatment strategies, but also opens up new avenues for future basic research on lncRNAs related to arginine methylation.

## Data Availability

The datasets presented in this study can be found in online repositories. The names of the repository/repositories and accession number(s) can be found in the article/**Supplementary Material**.

## Supplementary material

Supplementary Material 3 lncRNAs were associated with patient prognosis.
