# Supplementary figures and images for: A novel arginine methylation-associated lncRNA signature effectively predicts prognosis in breast cancer patients
Source: Front Oncol. 2024 Oct 1;14:1472434. doi: 10.3389/fonc.2024.1472434 (PMC11473254; doi:10.3389/fonc.2024.1472434)

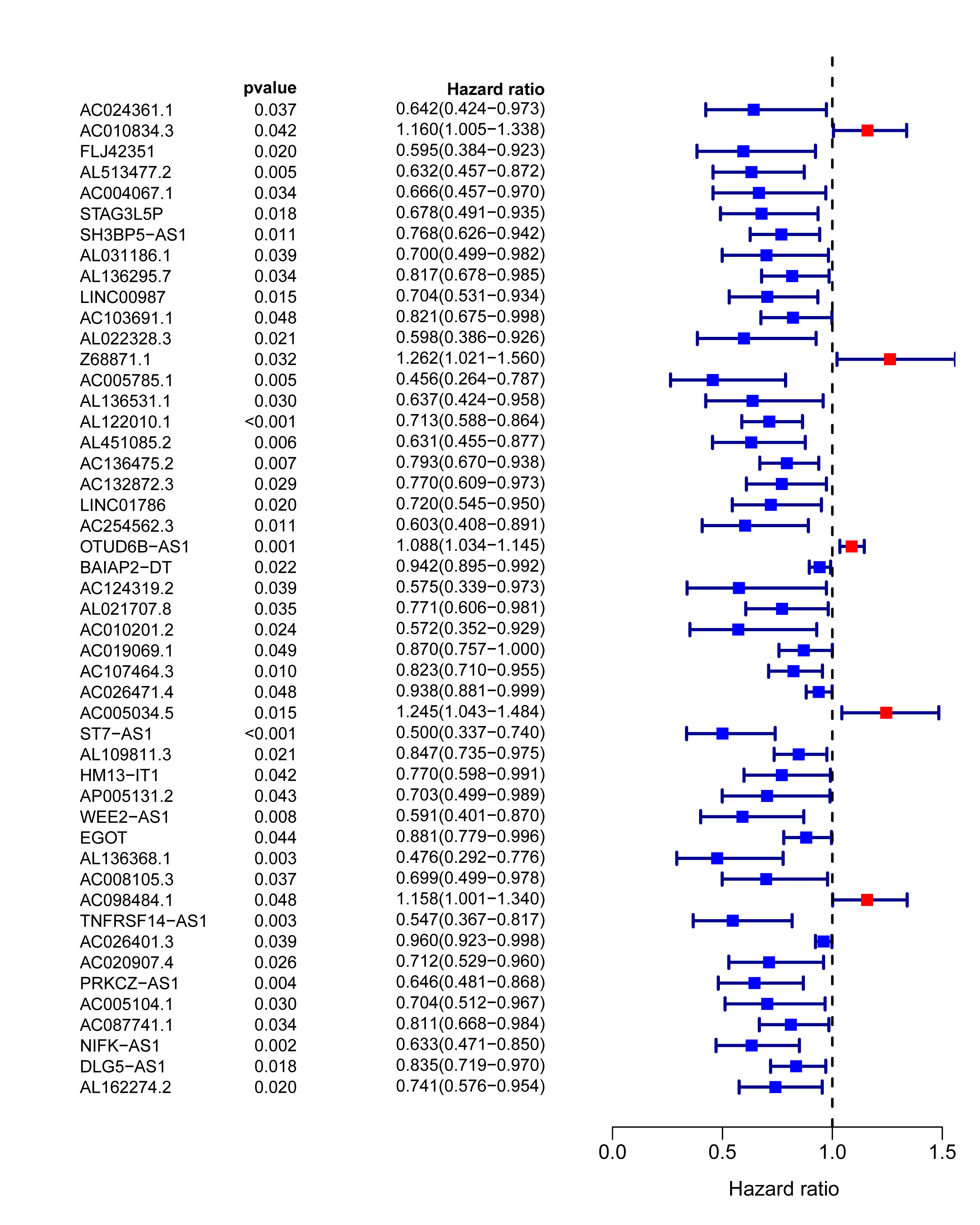

Supplement: Supplementary file 3 [file Image1.tif]
